# A novel approach for eliminating metal artifacts based on MVCBCT and CycleGAN

**DOI:** 10.3389/fonc.2022.1024160

**Published:** 2022-11-10

**Authors:** Zheng Cao, Xiang Gao, Yankui Chang, Gongfa Liu, Yuanji Pei

**Affiliations:** ^1^ National Synchrotron Radiation Laboratory, University of Science and Technology of China, Hefei, China; ^2^ Hematology and Oncology Department, Hefei First People’s Hospital, Hefei, China; ^3^ School of Nuclear Science and Technology, University of Science and Technology of China, Hefei, China

**Keywords:** tomography, MVCBCT, sCT CycleGAN, metal artifact reduction, radiotherapy dosimetry

## Abstract

**Purpose:**

To develop a metal artifact reduction (MAR) algorithm and eliminate the adverse effects of metal artifacts on imaging diagnosis and radiotherapy dose calculations.

**Methods:**

Cycle-consistent adversarial network (CycleGAN) was used to generate synthetic CT (sCT) images from megavoltage cone beam CT (MVCBCT) images. In this study, there were 140 head cases with paired CT and MVCBCT images, from which 97 metal-free cases were used for training. Based on the trained model, metal-free sCT (sCT_MF) images and metal-containing sCT (sCT_M) images were generated from the MVCBCT images of 29 metal-free cases and 14 metal cases, respectively. Then, the sCT_MF and sCT_M images were quantitatively evaluated for imaging and dosimetry accuracy.

**Results:**

The structural similarity (SSIM) index of the sCT_MF and metal-free CT (CT_MF) images were 0.9484, and the peak signal-to-noise ratio (PSNR) was 31.4 dB. Compared with the CT images, the sCT_MF images had similar relative electron density (RED) and dose distribution, and their gamma pass rate (1 mm/1%) reached 97.99% ± 1.14%. The sCT_M images had high tissue resolution with no metal artifacts, and the RED distribution accuracy in the range of 1.003 to 1.056 was improved significantly. The RED and dose corrections were most significant for the planning target volume (PTV), mandible and oral cavity. The maximum correction of Dmean and D50 for the oral cavity reached 90 cGy.

**Conclusions:**

Accurate sCT_M images were generated from MVCBCT images based on CycleGAN, which eliminated the metal artifacts in clinical images completely and corrected the RED and dose distributions accurately for clinical application.

## Introduction

Metal artifacts are a common problem in kilovoltage CT images and radiation therapy. In the process of CT scanning, when X-rays pass through metal implants, such as metal dentures and metal hip joints in patients, erroneous X-ray projections will be produced due to the combined effects of beam hardening, scattering, photon starvation, noise enhancement, volume effects and other factors (1, 2), resulting in bright and dark stripes and radial areas in the reconstructed images; these are known as metal artifacts. Metal artifacts not only affect the diagnosis and the accurate delineations of the tumour target volume and normal tissues but also introduce dose calculation errors in radiation therapy by reducing the accuracy of relative electron densities (RED), which endanger the efficacy and safety of radiotherapy for patients (3–5).

Traditional metal artifact reduction (MAR) algorithms mainly include the interpolation method and iterative method (6–8), which often introduce new artifacts into images, resulting in image distortion (9–12). In recent years, deep learning technology has developed rapidly and has been widely applied in the field of image processing; it has provided new ideas for MAR in CT images. Yu et al. combined the traditional MAR method with a convolutional neural network (CNN) and achieved a higher accuracy than the traditional MAR method (13). Zhang et al. corrected metal artifacts in cervical CT images by using a CNN-based method (14). Zhu et al. trained U-Net based on a digital anthropomorphic head phantom and verified its MAR effect through PMMA phantoms containing aluminium rods and copper rods (15). Wang et al. developed an interpretable network model named InDuDoNet by combining sinogram and image data and embedding imaging geometric constraints in training (16). Yu et al. also designed a new deep learning framework by combining the advantages of the sinogram and image learning to obtain MAR images through multiple filtered back-projection reconstruction of the sinogram (17).

All the above studies are supervised methods that require paired CT images with the same anatomical structure, one with and the other without metal artifacts, for model training. However, it is clinically impractical to obtain such pairs of images. To obtain paired data, some studies used simulated phantoms for model training (15), and other studies artificially generated metal artifacts on metal-free CT images through theoretical calculations (13, 14, 16, 17). A simulated phantom is very different from the real human body, and the artificially generated metal artifacts cannot accurately simulate the real physical mechanisms of CT imaging. Therefore, the above two methods have poor generalization ability to real patient data (13–17). To solve the problem of the lack of paired training data, Liao et al. proposed an unsupervised network model named ADN, which used unpaired data for training (18), and its generalization ability was significantly improved compared to the supervised models that used synthetic data. Nevertheless, metal artifacts on CT images of real patients are still clearly residual and cannot be completely eliminated

This study aims to completely eliminate metal artifacts in CT images based on paired data from real patients. Compared with CT images, MVCBCT images have higher noise and lower soft tissue resolution, but the higher X-ray energy greatly reduces the photon starvation and radiation hardening effects, making the metal artifacts almost negligible, and this feature can be applied to MAR in CT images (19–21). In this work, we proposed a novel MAR approach using paired MVCBCT images and planning CT images. First, paired metal-free MVCBCT (MV_MF) images and metal-free planning CT (CT_MF) images were used for training the cycle-consistent adversarial network (CycleGAN) model. Then, synthetic metal-free CT (sCT_MF) images were generated from MV_MF images in the test dataset and compared with CT_MF images in terms of image quality, the RED distributions of organs at risk (OARs) and the dose calculation in radiation therapy. Finally, metal cases were used to evaluate the effect of MAR. The synthetic metal-containing CT (sCT_M) images were generated from the metal-containing MVCBCT (MV_M) images and compared with metal-containing CT (CT_M) images. The comparison of sCT_M and CT_M images was implemented with imaging and dosimetry to evaluate the radiation dosimetry improvement in the generated sCT_M images.

## Materials and methods

**Figure 1:**
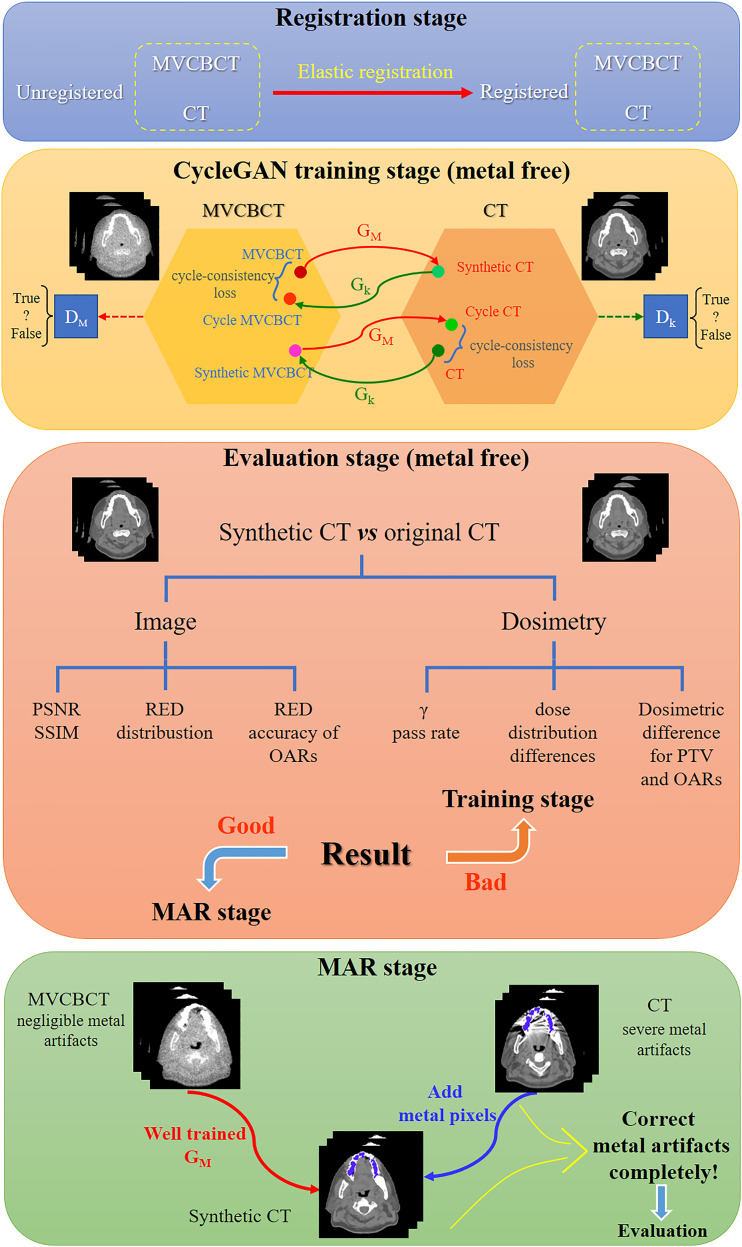
Schematic diagram of metal artifact correction based on MVCBCT and CycleGAN. The process of this research was divided into the registration stage, CycleGAN training stage, evaluation stage and MAR stage.

As illustrated in **Figure 1**, the process of this research was mainly divided into four stages. First, the CT images and MVCBCT images of the same patient were elastically registered in the registration stage. For metal-free images in the training set, CT numbers range from -1000 HU to 3000 HU for CT and from -1000 HU to 1400 HU for MV. Next, the CycleGAN model was trained using the metal-free images to generate sCT_MF images from MV_MF images. Then, in the third stage, the accuracy of the generated sCT_MF images was evaluated with imaging and dosimetry to judge whether the sCT images generated by the model were accurate enough to perform MAR. Finally, in the MAR stage, based on the well-trained CycleGAN model, metal-artifacts-free sCT_M images were generated from MV_M images; then, the metal pixels in the CT_M images were copied to the corresponding pixel positions in the sCT_M images. Specifically, the CT numbers in the MV_M images exceeding 1400 HU were modified to 1400 HU, and the sCT_M images without added metal pixels were generated through the CycleGAN model. In the works of Liao et al. and Wang et al., 2500 HU was used as the threshold of metal segmentation in CT images (16, 18). However, bright metal artifacts may still exist in the metal region segmented by this method. We observed that there is almost no metal artifact in the MVCBCT images and the CT number of metal is not less than 300 HU. Therefore, in order to reduce the metal artifacts contained in the segmented metal regions as much as possible, we identified the intersection regions with HU values greater than 2500 in the CT_M images and greater than 300 in the MV_M images as the metal regions. The final MAR images were obtained by copying the CT numbers of the metal pixels in the CT_M images into the previously generated sCT_M images.

### Data acquisition and preprocessing

Metal dentures have diverse materials and complex shapes. When their size is large or RED is high, severe metal artifacts appear in CT images, destroying the image quality and the accuracy of RED information. Therefore, the correction of metal artifacts caused by metal dentures has good clinical application value. In this study, CT and MVCBCT images of head cancer patients were obtained from the dataset.

Paired planning CT images and MVCBCT images of 126 patients without metal dentures and 14 patients with metal dentures were collected in this study, and the scans included the head. The CT images were derived from a Siemens SOMATOM Spirit helical CT scanner (tube voltage of 130 kV, slice thickness of 3 mm, 16-bit image output). The paired MVCBCT images were obtained in the first fraction (Siemens Artiste Medical Electron Linear Accelerator, 6 MV, 0.54 mm×0.54 mm×0.54 mm). The images from the temporomandibular joint to the mandible were selected for training and evaluation. The images from ninety-seven patients without metal in their scans were randomly selected for model training; these data included 1762 paired planning CT slices and MVCBCT slices. The remaining images of the 29 metal-free patients (457 slices) were used as the metal-free test set, and the 14 patients with metal dentures (86 slices) were used as the metal test set.

Data preprocessing was required before model input. First, the Elastix multiresolution B-spline registration method (22, 23) was used to elastically align the CT images and MVCBCT images of the same patient. Then, the images were resampled to 1 mm×1 mm and cropped to 256×256 pixels. Then, the hyperbolic tangent function (Tanh) was used to scale the CT values to (–1,1), and is defined as $\text{Tanh}\left(\text{x}\right)\;=\;\frac{{\text{e}}^{x}-{\text{e}}^{-x}}{{\text{e}}^{x}+{\text{e}}^{-x}}$ . Before being processed by Tanh, the HU values of the CT images and MVCBCT images were scaled linearly with three methods as follows:

1) *X*(*MVCBCT*) = *Tanh*(*HU*(*MVCBCT*)/400) and *X*(*CT*) = *Tanh*(*HU*(*CT*)/400).

2) *X*(*MVCBCT*) = *Tanh*(*HU*(*MVCBCT*)/150) and *X*(*CT*) = *Tanh*(*HU*(*CT*)/300).

3) *X*(*MVCBCT*) = *Tanh*(*HU*(*MVCBCT*)−800/400) and *X*(*CT*) = *Tanh*(*HU*(*CT*)−1600/960).

Processed by the above three methods, these data were used for model training separately to obtain three groups of results, named P1, P2 and P3.

### CycleGAN-based unsupervised model

Although the paired CT and MVCBCT images were selected as training data, there were still problems in supervised pixel-to-pixel learning. The setup error between the two scans, the differences in the mouth opening size and image distortions caused by elastic registration may introduce differences into the CT images and MVCBCT images. Therefore, this study used CycleGAN for unsupervised learning because pixel-level correspondence is not necessary.

Generative adversarial networks (GANs) are unsupervised deep learning models that mainly include a generator (G) and a discriminator (D). A trained GA-B could generate image A’, which has the structure of image A and the style of image B. CycleGAN models (24) include two generators and two discriminators and add cycle-consistency loss for training. CycleGAN has been widely used for interconversion between different types of medical images (25–30). The structure of CycleGAN used in this study is consistent with that reported in the literature (24), and the model structure is shown in **Figure 1**. ResUNet (31) was used as the generator, and the Adam optimizer was selected to train the model with a batch size of 6 on one NVIDIA Quadro RTX 6000 GPU. The learning rate was constant at 0.0002 for the first 100 epochs of training and attenuated by 1% per epoch for the last 100 epochs. A previous study showed that paired data have better performance than unpaired data when using CycleGAN to generate sCT (32). Therefore, this study used deformation-registered paired data for training.

### Imaging evaluation

Compared to the planning CT images, the image quality of synthetic CT images was evaluated by the peak signal-to-noise ratio (PSNR) and structural similarity (SSIM) index.


(1) $$PSNR\left(I_{1},I_{2}\right)=10\times \log_{10}(\frac{MAX^{2}}{RMSE{\left(I_{1},I_{2}\right)}^{2}})$$

(2) $$SSIM\left(I_{1},I_{2}\right)=\frac{\left(2\mu_{I_{1}}\mu_{I_{2}}+c_{1}\right)\left(2\sigma_{I_{1},I_{2}}+c_{2}\right)}{(\mu_{I_{1}}^{2}+\mu_{I_{2}}^{2}+c_{1})\left(\sigma_{I_{1}}^{2}+\sigma_{I_{2}}^{2}+c_{2}\right)}$$

To compute the dose using CT images in photon radiotherapy, the CT numbers need to be converted to RED values through the CT-ED conversion curve. Since the CT-ED curves are very different between CT images and MVCBCT images, it is necessary to compare their RED values rather than their CT numbers. The CIRS 062 electron density phantom was used to obtain CT numbers corresponding to the RED values in the range of 0 to 1.456. The correspondence between CT numbers and RED values of different metals was obtained through the head part of a CIRS ATOM 701-B dosimetry anthropomorphic phantom with aluminium alloy (RED: 2.43), titanium alloy (RED: 3.73) and stainless steel (RED: 6.83) plugs.

In addition, the RED distributions of OARs in CT images were analysed. The main OARs affected by metal artifacts, such as the mandible, oral cavity, parotid gland and spinal cord, were delineated, and their RED distributions were compared with those in the MVCBCT images and sCT images.

### Dosimetry evaluation

The target volume was redelineated according to the anatomical structure of each patient in the test set with reference to the actual target volume position of NPC patients. In the treatment planning system (TPS), the same prescription dose (PTV: 6000 cGy) was used to produce a dynamic intensity-modulated plan (Eclipse 15.6, AXB algorithm) on the CT images, and then the plan was copied to the corresponding sCT images. Finally, the global gamma pass rates and the three-dimensional dose distribution difference of the target area and the OARs were compared. The gamma pass rates between the radiotherapy plans of sCT images and CT images were calculated using PTW Verisoft software, version 6.0 (PTW, Frieburg, Germany), and the criteria included 2 mm/2% and 1 mm/1% (distance error/dose error), respectively. V95%, V100%, V110% (Vx% means the percentage of volume receiving at least x% of the prescription dose), D5, D95 (Dx means the doses to x% of the volume), Dmean (mean dose of the volume) for the PTV, D2 and Dmean for the mandible, D50 and Dmean for the oral cavity and parotid gland, and D0.1 cc (dose to 0.1 cc volume) for the spinal cord were investigated.

The significance test of the RED and dosimetry data was performed using IBM SPSS Statistics 26 software. Paired and unpaired t tests were used for normally distributed data, and the Mann-Whitney U test was used for nonnormally distributed unpaired data (33).

## Results

**Figure 2:**
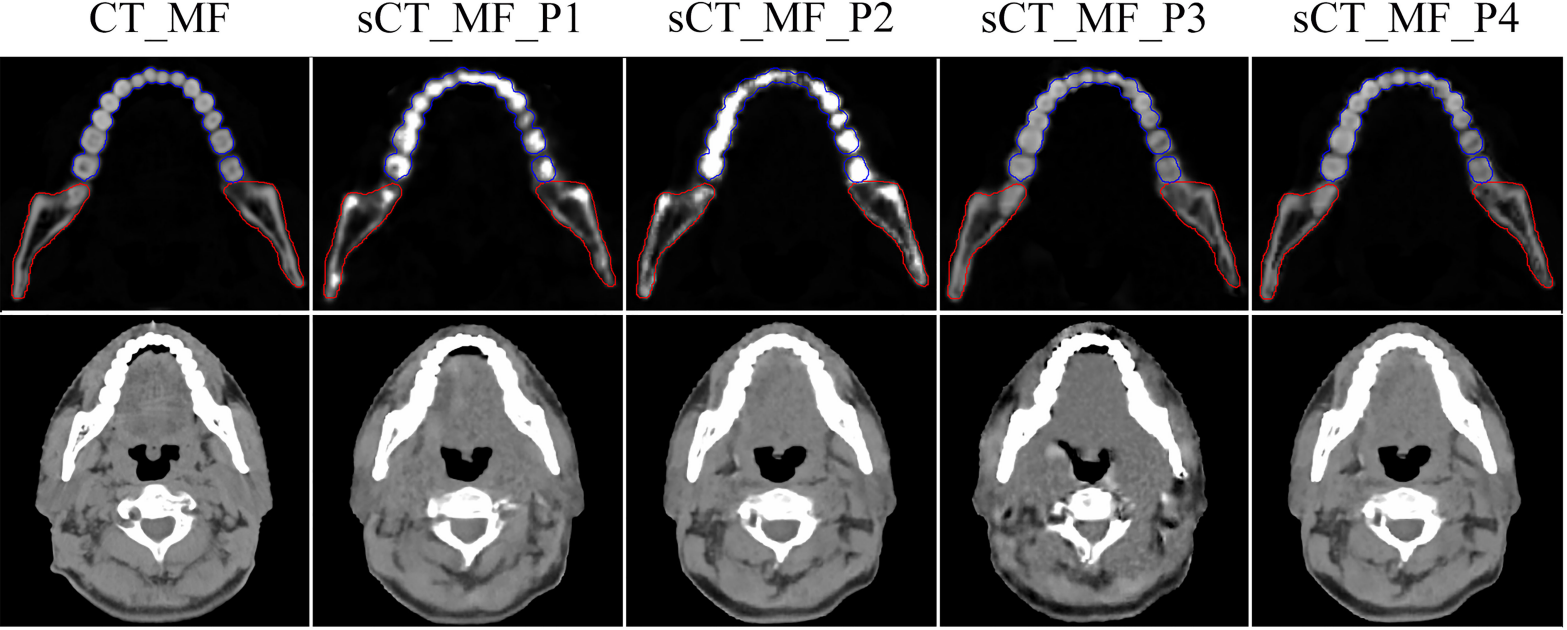
Visualized differences of CT_MF and sCT_MF images with different preprocessing methods. The display windows for the first and second rows were [0, 3000] HU and [-360, 440] HU, respectively. Blue lines represent the contour of the teeth, and red lines represent the contour of the mandible. The images in the first to fifth columns were CT_MF, sCT_MF_P1, sCT_MF_P2, sCT_MF_P3 and sCT_MF_P4, respectively. Different image preprocessing methods have their own advantages in different CT number ranges, and sCT_MF_P4 performs best.

**Figure 2** shows the effects of different preprocessing methods (P1, P2 and P3) on sCT image quality. For organs such as the mandible and teeth, more uniform CT numbers and higher similarity with the CT_MF images were achieved using sCT_ MF_P3 compared with sCT_MF_P1 and sCT_MF_P2. The CT numbers of teeth for CT_MF, sCT_MF_P1, sCT_MF_P2 and sCT_MF_P3 were 1473 ± 554 HU, 1726 ± 863 HU, 2003 ± 995 HU and 1476 ± 481 HU, respectively. The CT numbers of the mandible were 838 ± 494 HU, 891 ± 631 HU, 917 ± 417 HU and 848 ± 425 HU, respectively. **Table 1** shows a comparison of the accuracy of sCT_MF images with different preprocessing methods in the ranges of [-200, 400] HU, [400, 800] HU, [800, 3000] HU and [-1000, 3000] HU. sCT_MF_P2 performed best at [-200, 400] HU, sCT_MF_P1 performed best at [400, 800] HU, and sCT_MF_P3 performed best at [800, 3000] HU. Obviously, different image preprocessing methods have their own advantages in different CT number ranges. Therefore, the three trained models with different preprocessing methods were combined to produce the new sCT_MF (sCT_MF_P4), which used the part of sCT_MF_P2 below 400 HU, the part of sCT_MF_P1 at [400, 800] HU and the part of sCT_MF_P3 over 800 HU. The accuracy of the sCT_MF_P4 image was improved significantly (PSNR: 31.4 ± 1.3 dB; SSIM: 0.9484 ± 0.0090). It should be noted that the generated sCT_MF and sCT_M images in the following were processed by the combined P4 method.

**Table 1:** The evaluation of sCT_MF images with different preprocessing methods (PSNR (dB)/SSIM).

CT number range(HU)	sCT_MF_P1	sCT_MF_P2	sCT_MF_P3	sCT_MF_P4
-1000~3000	30.0/0.9459	28.2/0.9435	30.7/0.9345	**31.4/0.9484**
-200~400	23.9/0.8599	**24.2/0.8689**	22.6/0.8395	/
400~800	**22.8/0.9558**	22.2/0.9534	21.4/0.9470	/
800~3000	27.9/0.9652	24.9/0.9555	**33.0/0.9701**	/

sCT_MF_P1, metal-free sCT images obtained by the prepossessing method named P1; sCT_MF_P2, metal-free sCT images obtained by the prepossessing method named P2; sCT_MF_P3, metal-free sCT images obtained by the prepossessing method named P3; metal-free sCT images obtained by the combined prepossessing method named P4. The best PSNR and SSIM values in different HU ranges of sCT_MF images are marked in bold.

**Figure 3:**
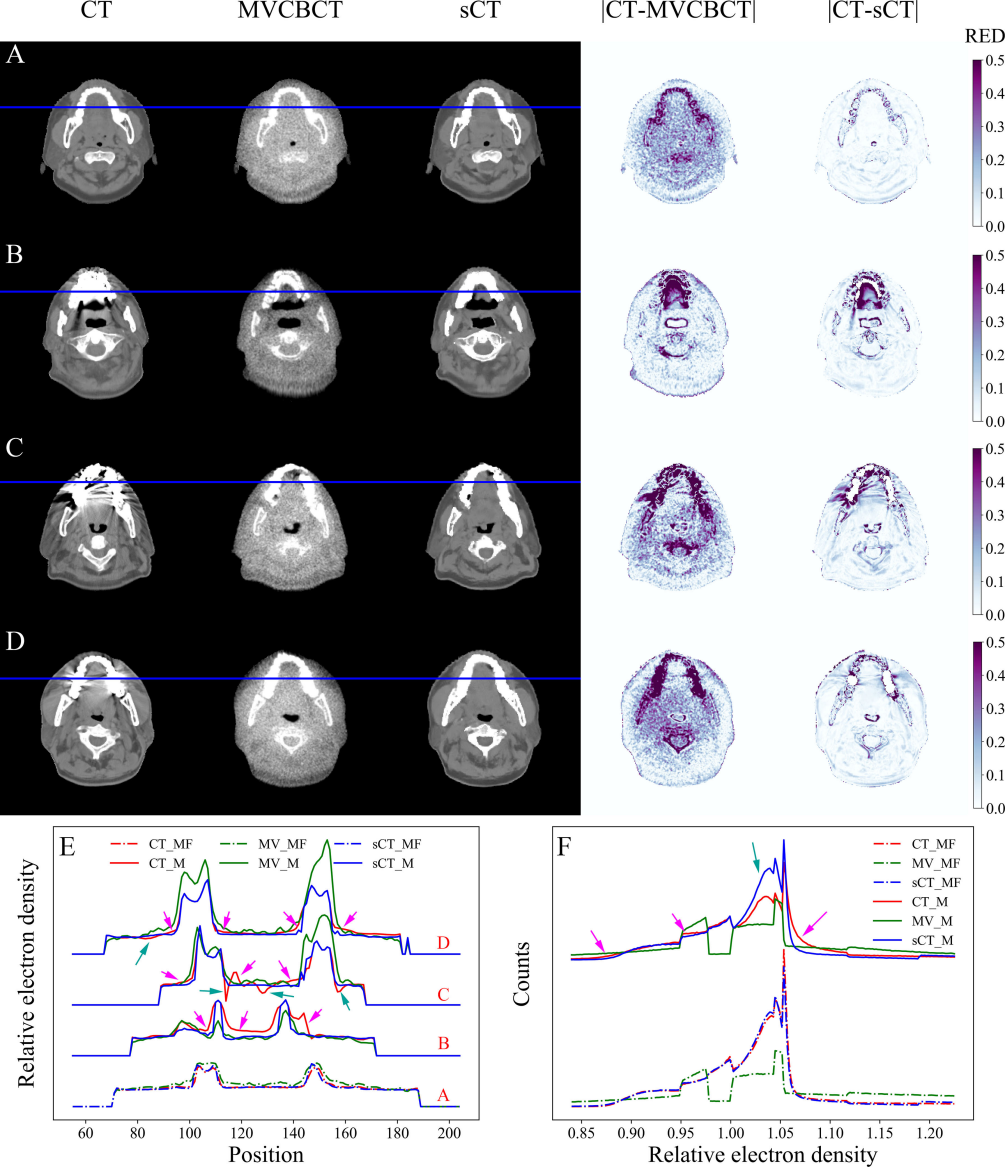
RED comparison of CT, MVCBCT and sCT images. **(A)** Metal-free images. **(B-D)** Metal-containing images. **(E)** RED distribution curves for the blue lines in **(A–D)**. **(F)** RED histograms of the images. The display window for CT, MVCBCT and sCT images was [-360, 440] HU. The RED distributions of CT_MF and sCT_MF images were almost coincident, and the metal artifacts were completely eliminated in sCT_M images after the MAR stage.

**Figure 4:**
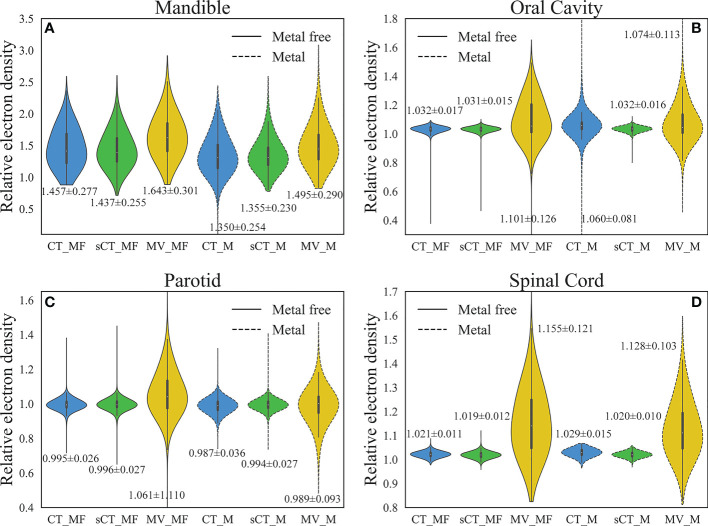
Comparison of RED distributions for OARs. **(A)** Mandible. **(B)** Oral cavity. **(C)** Parotid. **(D)** Spinal cord. The numbers marked in the figure are the average ± standard deviation. The RED values of the main OARs in sCT_MF images were accurate, and the inaccurate RED values caused by metal artifacts in CT_M images were corrected in sCT_M images after the MAR stage.

The RED comparison of CT, MVCBCT and sCT images is shown in **Figure 3**. In **Figure 3A**, the difference in the RED values of CT_MF and sCT_MF images was significantly smaller than that of CT_MF and MV_MF images, especially in soft tissues. **Figure 3F** and part A in **Figure 3E** show that the RED curves of CT_MF and sCT_MF images were almost coincident, while the curves of CT_MF and MV _MF images were quite different. The RED distributions of OARs for CT_MF and sCT_MF images were almost the same (**Figure 4**), and the difference was not statistically significant (P > 0.05 in **Table 2**). Compared with the large difference in the RED values of CT_MF and MV_MF images, the RED values of the main OARs in sCT_MF images were sufficiently accurate to be used for radiotherapy dose calculations.

**Table 2:** P value comparison of RED distributions for OARs.

OARs	CT_MF vs. sCT_MF^a^	CT_M vs .sCT_M^a^	CT_MF vs .CT_M^b^	CT_MF vs .sCT_M^b^
Mandible	0.055	0.813	< 0.001^*^	< 0.001^*^
Oral Cavity	0.805	< 0.001^*^	< 0.001^*^	0.509
Parotid	0.499	0.174	0.355	0.744
Spinal Cord	0.056	0.006^*^	< 0.001^*^	0.512

a: Paired-sample T test. b: Independent-sample T test.*: Statistically significant differences (P< 0.05). RED, Relative electron density; OARs, organs at risk; CT_MF, metal-free CT images; CT_M, metal-containing CT images; sCT_MF, metal-free sCT images; sCT_M, metal-containing sCT images.

**Figure 5:**
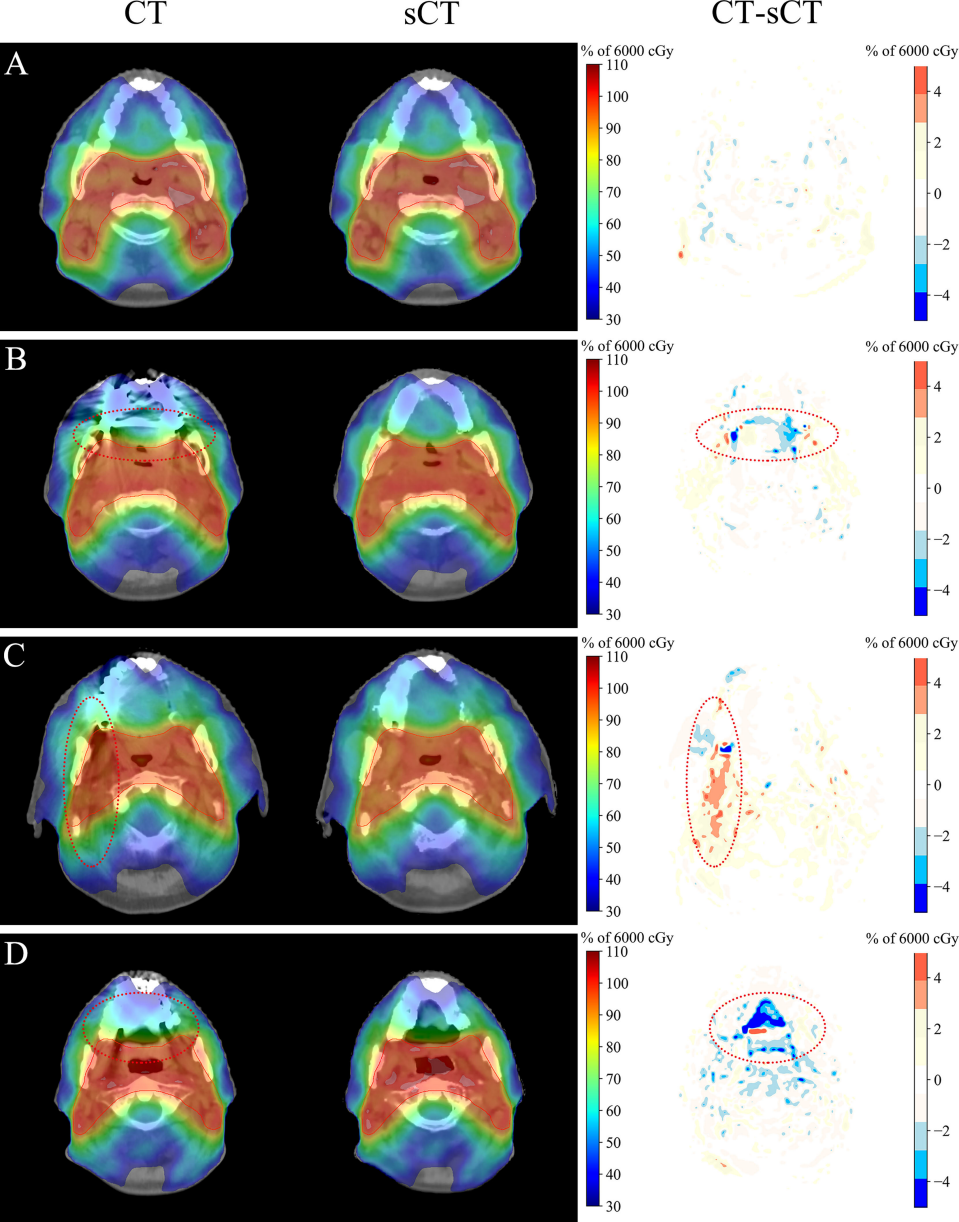
Dosimetric comparison of CT and sCT images. **(A)** Metal-free images. **(B–D)** Metal-containing images. The display window for the CT and sCT images was [-360, 440] HU. The dose distribution of sCT_MF images was consistent with that of CT_MF images, and there were obvious dose differences between CT_M and sCT_M images in the area with serious metal artifacts.

**Figure 6:**
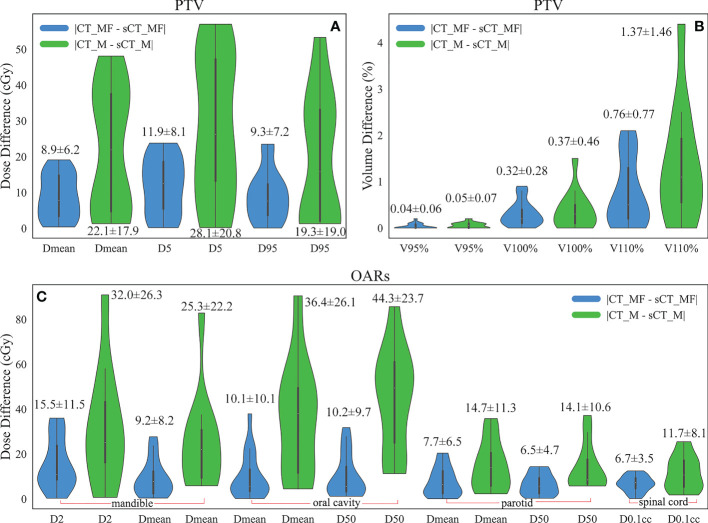
Comparison of the absolute dose errors for the PTV and OARs. **(A)** Dose difference (cGy) of the PTV. **(B)** Volume difference (%) of the PTV. **(C)** Dose difference (cGy) of the OARs. The numbers marked in the figure are the average ± standard deviation. Compared with metal-free cases, the average and standard deviation of the dose differences for the PTV and OARs doubled for cases with metal artifacts.

The dose distributions based on CT_MF and sCT_MF images were slightly different, as shown in **Figure 5A**. The gamma pass rates of the sCT_MF-based plans were 99.72% ± 0.29% (2 mm/2%) and 97.99% ± 1.14% (1 mm/1%) compared to the CT_MF-based plans. The blue part in **Figure 6** shows the absolute dose errors of CT_MF and sCT_MF images, which were 8.9 ± 6.2 cGy, 11.9 ± 8.1 cGy, 9.3 ± 7.2 cGy, 0.04% ± 0.06%, 0.32% ± 0.28% and 0.76% ± 0.77% for Dmean, D5, D95, V95, V100%, and V110% of the PTV, respectively. For the mandible (D2 and Dmean) and oral cavity (D50 and Dmean), the maximum differences were all less than 40 cGy, and the average difference was approximately 10 cGy. For the parotid (D50 and Dmean) and spinal cord (D0.1 cc), the max differences were all less than 20 cGy, and the average difference was approximately 7 cGy. The above results demonstrate that the dose distribution of sCT_MF images was consistent with that of CT_MF images, which proves the accuracy of our proposed method for generating synthetic CT images from MVCBCT images.

LI (34) and NMAR (35) are widely used approaches to MAR. **Supplementary figure 1** and **Supplementary figure 2** show the qualitative comparisons of our MAR method with the LI and NMAR methods on the clinical data and phantom data, respectively. It is clear that our method completely eliminates metal artifacts in both clinical data and phantom data, whereas both the LI and NMAR methods not only fail to completely eliminate metal artifacts, but also create a large number of new artifacts in the images. For MAR of CT_M images, metal artifacts with varying severities were completely removed from sCT_M images (**Figures 3B–3D**). The sCT_M images had comparable quality to metal-artifact-free CT_MF images. Notably, according to the RED differences in **Figures 3B–3D**, the RED information corrupted by metal artifacts was corrected in the sCT_M images, and the RED difference in the area away from the metal artifacts was very small. It was evident from **Figure 3F** and parts B-D in **Figure 3E** that the difference in the RED values of the CT_M and sCT_M images was larger than that of the CT_MF and sCT_MF images. In **Figure 4**, the RED values of the CT_M and sCT_M images were 1.350 ± 0.254 and 1.355 ± 0.230 for the mandible (P = 0.813), 1.060 ± 0.081 and 1.032 ± 0.016 for the oral cavity (P< 0.001), 0.987 ± 0.036 and 0.994 ± 0.027 for the parotid (P = 0.174), and 1.029 ± 0.015 and 1.020 ± 0.010 for the spinal cord (P = 0.006), respectively.

The dose distributions based on CT_M and sCT_M images are shown in **Figures 5B–5D**, and the gamma pass rates of the sCT_M-based plans were 99.55% ± 0.35% (2 mm/2%) and 96.55% ± 1.54% (1 mm/1%) compared to the CT_M-based plans. The green part in **Figure 6** shows the absolute dose errors from the CT_M and sCT_M images, which were 22.1 ± 17.9 cGy, 28.1 ± 20.8 cGy, 19.3 ± 19.0 cGy, 0.05% ± 0.07%, 0.37% ± 0.46% and 1.37% ± 1.46% for Dmean, D5, D95, V95%, V100%, and V110% of the PTV, respectively. For the PTV (Dmean, D5), mandible (Dmean), oral cavity (D50 and Dmean) and parotid (D50 and Dmean), the absolute dose errors of the sCT_M and CT_M images were statistically significant compared to the absolute errors of the sCT_MF and CT_MF images (**Figure 6C** and **Table 3**). The dose difference in the spinal cord far away from metal artifacts was not statistically significant (6.7 ± 3.5 vs. 11.7 ± 8.1, P > 0.05 in **Table 3**).

**Table 3:** The difference significance test between the absolute dose errors of sCT_M and CT_M images and the absolute errors of sCT_MF and CT_MF images for the PTV and OARs.

Structures	Dosimetry Parameter	Test Method	P Value
PTV	Dmean	T	0.047^*^
	D5	T	0.038^*^
	D95	T	0.142
	V95%	U	0.487
	V100%	U	0.781
	V110%	U	0.517
Mandible	D2	U	0.089
	Dmean	U	0.009^*^
Oral Cavity	Dmean	U	0.002^*^
	D50	U	< 0.001^*^
Parotid	Dmean	T	0.047^*^
	D50	U	0.036^*^
Spinal Cord	D0.1 cc	T	0.079

T: Independent-sample T test for normally distributed data. U: Independent-sample Mann-Whitney U Test for nonnormally distributed data. *: Statistically significant differences (P< 0.05). PTV, planning target volume; OARs, organs at risk; CT_MF, metal-free CT images; CT_M, metal-containing CT images; sCT_MF, metal-free sCT images; sCT_M, metal-containing sCT images; Vx%, the percentage of volume receiving at least x% of the prescription dose; Dx, the doses to x% of the volume; Dmean, mean dose of the volume; D0.1 cc, dose to 0.1 cc volume.

## Discussion

In this study, a novel approach, in which the advantages of the CycleGAN model and the characteristics of negligible metal artifacts in MVCBCT images were integrated, was proposed to address the MAR task. The results suggested that our proposed method could be used to completely remove metal artifacts in original CT images and correct the destroyed RED distributions, and hence a more accurate dose calculation for radiotherapy can be produced.

Different normalization methods in preprocessing could affect the accuracy of sCT images, as shown in **Figure 2**. The difference between the P1, P2 and P3 methods was mainly because the main range of the CT numbers involved in the training stage varied with the preprocessing methods. Therefore, the three trained models with different preprocessing methods were combined to produce the final sCT images.

TPS requires images to be calibrated for RED values before dose calculations are performed (36). Considering the large gap between the CT-ED curves of CT and MVCBCT images (19), it is not intuitive to directly compare the difference in CT numbers when evaluating image quality in the study by Zhao et al. (37). Therefore, our image evaluation approach mainly focused on the RED values.

In the results, we analysed the image quality and dose calculation accuracy of the generated sCT images for the test sets with and without metal. Since we cannot obtain ground truth images for the clinical metal-containing images, in previous studies, quantitative evaluation could only be performed on synthetic data or simulated phantoms (13–18). In our study, we indirectly realized the quantitative evaluation of the MAR effect on clinical images through the quantitative evaluation of the sCT_MF images and the statistical analysis of sCT_MF and sCT_M images. The model was trained with paired CT_MF and MV_MF images, and CT_MF and sCT_MF images had high consistency in terms of image quality, RED values and dose distributions. The PSNR and SSIM values for the CT_MF and sCT_MF images comparison were 31.4 ± 1.3 and 0.9484 ± 0.0090, respectively, which are comparable to Liang et al.’s study (30.65 ± 1.36/0.85 ± 0.03), Vinas et al.’s study (29.7 ± 2.7/0.927 ± 0.028), Harms et al.’s study (PSNR: 32.3 ± 5.9) and Chen et al.’s study (30.75 ± 3.89/0.9642 ± 0.0186) for head patient images (25, 27, 38, 39). The gamma pass rates (1 mm/1%) of the sCT_MF-based plans (97.99% ± 1.14%) were better than those obtained in Liang et al.’s study (96.26% ± 3.59%) and Li et al.’s study (95.5% ± 1.6%) (25, 40). Therefore, we believe that the sCT_M images generated from MV_M images were sufficiently accurate to evaluate the effect of MAR.

In previous studies, the excellent MAR performance on simulated images could not be sustained on clinical images. Qualitative analyses showed that artifacts remained in images after MAR, and the image quality was also degraded (13, 14, 16–18). In contrast, metal artifacts in clinical images were eliminated completely in our study (**Figure 3**). Furthermore, quantitative assessments of the MAR effect on clinical images were performed. The MV_M images were almost identical to the MV_MF images since the metal artifacts were barely visible (**Figures 3B–3D**). In the soft tissue region near the teeth, the RED distribution curves of CT_MF images were smooth, while those of CT_M images were not (**Figure 3E**). Some RED values were high (pink arrows) in CT_M images due to bright metal artifacts, while others (green arrows) were low due to dark metal artifacts. On the other hand, the curves in the corresponding areas in sCT_M images were as smooth as those in CT_MF images, which means that the RED values were accurately corrected in sCT_M images. In **Figure 3F**, the RED histograms of the CT_M and sCT_M images had obvious differences, especially in the RED value range of 1.003 to 1.056 (green arrow). This may be because metal artifacts mainly destroy RED values in the range of 1.003 to 1.056, while the damage is corrected in sCT_M images.

The RED distributions of OARs were different because of the different distances from the metal artifacts. Influenced by the metal artifacts, there were many pixels with low RED values in the mandible of CT_M images, and these pixels were corrected in the sCT_M images (**Figure 4A**). Due to the proximity to metal dentures, the RED values of the oral cavity in CT_M images were greatly affected by the metal artifacts (CT_MF vs. CT_M, P< 0.001 in **Table 2**), with a significantly higher mean and standard deviation (**Figure 4B**) and a large number of outliers that were too high or too low. The RED values of the oral cavity were almost consistent in the sCT_M and CT_MF images (P = 0.509 in **Table 2**), and there was a significant difference in the values of CT_M and sCT_M images (P< 0.001 in **Table 2**), which further proved the accuracy of RED correction for the oral cavity in sCT_M images. As shown in **Table 2**, the spinal cord and oral cavity had similar significance test results, which also proves that the RED values of the spinal cord were accurately corrected in sCT_M images.

For dose calculation, the gamma pass rates of sCT_M and CT_M images were lower than those of sCT_MF and CT_MF images, which was the results of MAR. As shown in **Figures 5B–5D**, there were obvious dose differences between CT_M and sCT_M images in the area with serious metal artifacts (elliptical dotted lines), and the maximum correction of the point dose could reach more than 5% of the total dose. Compared with metal-free cases, the average and standard deviation of the dose differences for the PTV and OARs doubled for cases with metal artifacts (**Figure 6**). The accuracy of the sCT_M-based dose calculation showed statistically significant improvements in the PTV and OARs (**Table 3**).

Finally, there are some works that need to be improved. The RED difference of the bone and tooth areas of the CT_MF and sCT_MF images was significantly greater than that of soft tissues. The results showed that the combination of multiple preprocessing methods could improve the accuracy of sCT images with high RED values, and this will be further researched in our next work.

## Conclusion

We proposed a novel MAR approach to complete the MAR task. In this approach, the advantages of the CycleGAN model and the characteristics of negligible metal artifacts in MVCBCT images are integrated. The model was trained on paired metal-free CT and MVCBCT images and generated metal-artifacts-free sCT images from metal-containing MVCBCT images to convert the task of MAR to the task of generating sCT images from MVCBCT images. The metal artifacts were completely removed in the sCT_M images, and the inaccurate RED values were corrected, which could significantly improve the accuracy of disease diagnosis and radiotherapy dose calculation.

## Data availability statement

The original contributions presented in the study are included in the article/**Supplementary Material**. Further inquiries can be directed to the corresponding authors.

## Author contributions

ZC conceived the experiments. XG collected the clinical dataset. ZC, GL and YP designed the study and analyzed the result. ZC, XG, YC, GL and YP participated in writing manuscript. All authors contributed to the article and approved the submitted version.

## Funding

Hefei Municipal Health Commission (CN) Applied Medicine Research Project (key project: hwk2018zd012)

## Conflict of interest

The authors declare that the research was conducted in the absence of any commercial or financial relationships that could be construed as a potential conflict of interest.

## Publisher’s note

All claims expressed in this article are solely those of the authors and do not necessarily represent those of their affiliated organizations, or those of the publisher, the editors and the reviewers. Any product that may be evaluated in this article, or claim that may be made by its manufacturer, is not guaranteed or endorsed by the publisher.
