# Supplementary material for: A novel approach for eliminating metal artifacts based on MVCBCT and CycleGAN
Source: Front Oncol. 2022 Nov 10;12:1024160. doi: 10.3389/fonc.2022.1024160 (PMC9686009; doi:10.3389/fonc.2022.1024160)
Supplement: Supplementary file 1 [file DataSheet_1.pdf]

Supplementary Material

1 Supplementary Figures

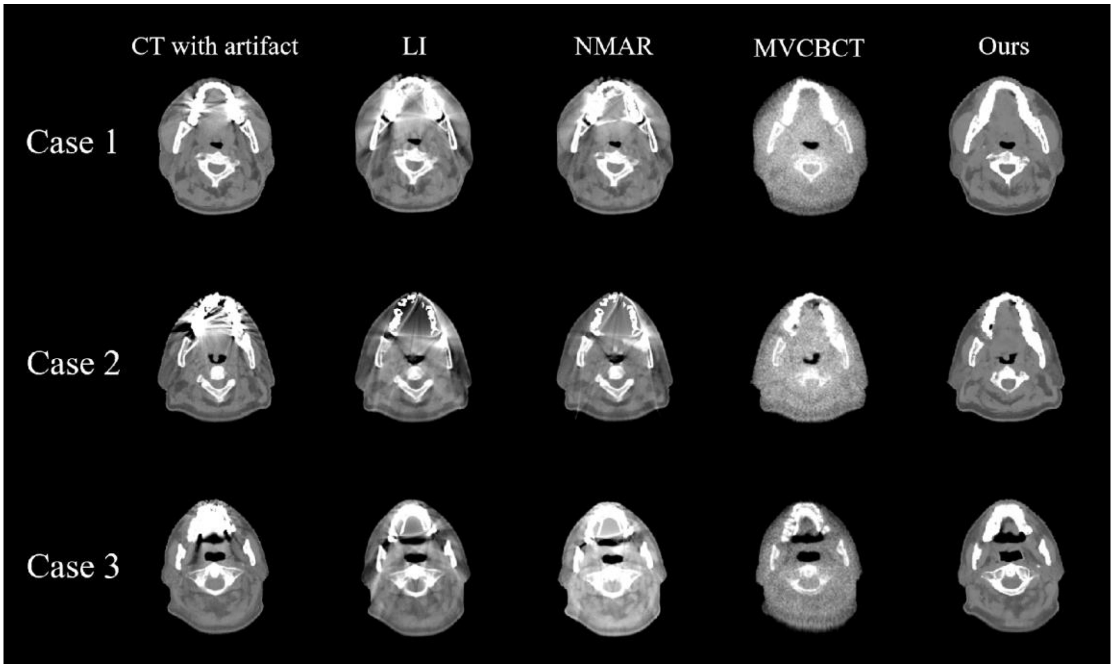

Supplementary Figure 1. Qualitative comparison with LI and NMAR methods on the clinical data. The first to fifth columns are (1) the original CT images with metal, (2) the images corrected by LI, (3) the images corrected by NMAR, (4) the MVCBCT images of the same patients at the same slices after elastic alignment, and (5) the images corrected by our MAR method, respectively. Our MAR method completely eliminates metal artifacts in clinical images, whereas both the LI and NMAR methods not only fail to completely eliminate metal artifacts, but also create a large number of new artifacts in the images.

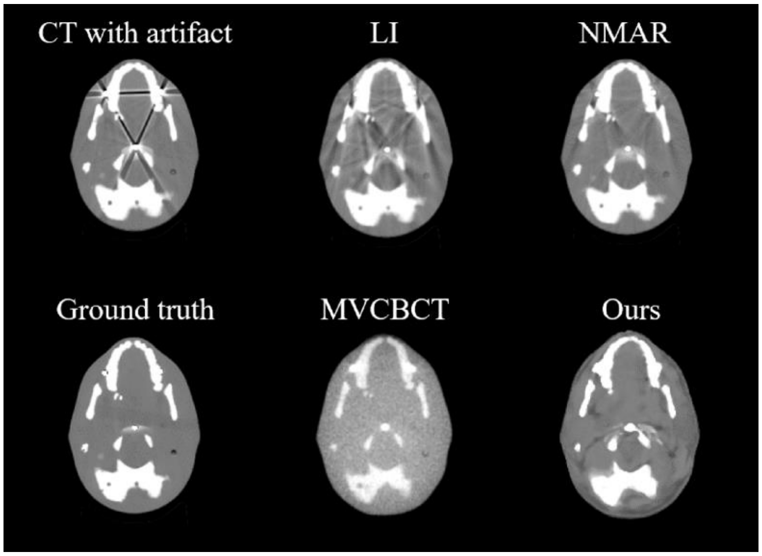

Supplementary Figure 2. Qualitative comparison with LI and NMAR methods on the phantom data. The phantom is CIRS ATOM 701-B dosimetry anthropomorphic phantom with 5 mm diameter stainless steel plugs. The ground truth images are obtained by adding metal pixels to the CT images of the phantom without metal. Both the ground truth images and the MVCBCT images are elastic aligned with the CT images containing metal artifacts. The phantom images are homogeneous whereas the clinical CT images are not. The generalisation ability of deep learning models is based solely on the amount and diversity of training data. As the differences between the phantom images and the clinical CT images used for training are huge, an overfitting phenomenon occurs when the trained model is applied to the CT images of the phantom. The pseudo-CT images generated by our MAR method based on the MVCBCT images of the phantom show structures resembling soft tissues of the human body, which is a typical overfitting phenomenon. Despite the overfitting phenomenon, the images corrected by our MAR method are completely free of metal artifacts compared to those corrected by the LI and NMAR methods, which still have a large number of secondary artifacts.
